# Bleomycin Loaded Magnetite Nanoparticles Functionalized by Polyacrylic Acid as a New Antitumoral Drug Delivery System

**DOI:** 10.1155/2013/462589

**Published:** 2013-08-05

**Authors:** Yue Xu, Yi Lin, Lin Zhuang, Jiong Lin, Jiahong Lv, Qin Huang, Jiadong Sun

**Affiliations:** ^1^Department of Orthodontics, Guanghua School of Stomatology, Hospital of Stomatology, Sun Yat-sen University, Guangdong Provincial Key Laboratory of Stomatology, Guangdong, Guangzhou 510055, China; ^2^State Key Laboratory of Optoelectronic Materials and Technologies, Guangdong Provincial Key Laboratory of Photovoltaics Technologies, School of Physics and Engineering, Institute for Solar Energy Systems, Sun Yat-sen University, Guangzhou 510275, China

## Abstract

*Objective*. To prepare, characterize, and analyze the release behavior of bleomycin-loaded magnetite nanoparticles (BLM-MNPs) coated with polyacrylic acid (PAA) as a new drug delivery system that can be specifically distributed in the tumor site. *Methods*. BLM-MNPs coated with PAA were prepared using a solvothermal approach. The particles were characterized using scanning electron microscope (SEM), vibrating sample magnetometer (VSM), and Fourier transform infrared spectroscopy (FTIR). The loading and release behaviors of BLM-MNPs were examined by a mathematical formula and in vitro release profile at pH 7.5. *Results*. The sphere Fe_3_O_4_ nanoparticles with the size of approximately 30 nm exhibit a saturation magnetization of 87 emu/g. The noncoordinated carboxylate groups of PAA confer on the good dispersibility in the aqueous solution and lead to a good loading efficiency of BLM reaching 50% or higher. Approximately 98% of immobilized BLM could be released within 24 h, of which 22.4% was released in the first hour and then the remaining was released slowly and quantitatively in the next 23 hours. *Conclusion*. BLM-MNPs were prepared and characterized successfully. The particles show high saturation magnetization, high drug loading capacity, and favorable release property, which could contribute to the specific delivery and controllable release of BLM, and the BLM-MNPs could be a potential candidate for the development of treating solid tumors.

## 1. Introduction

Bleomycin (BLM) is a glycopeptides antibiotic originally produced by the bacterium *Streptomyces verticillus*, acting by inducing the breakage of DNA strand [1]. It is commonly used either as a single agent or in combination with other chemotherapeutic regimens in the treatment of solid tumors, like squamous cell carcinomas [2], hemangioma [3], and Hodgkin's lymphoma [4]. However, dose-limiting toxic side effects such as pulmonary fibrosis [5], impaired lung function [6], and Reynaud's phenomenon [7] limit the clinical application of BLM, owing to nonspecific distribution to healthy normal tissues. Therefore, studies over the past few decades have focused on the development of drug delivery systems and administration routes for BLM to improve the treatment efficacy and reduce unwanted side effects [8–10]. 

Although intratumoral administration is a promising approach for the treatment of various solid tumors with minimal systemic toxicity, its efficacy is highly dependent on the timing and frequency of the drug injections because of its rapid clearance from the tumor site. It is proposed that a drug delivery system is required to ensure that the drug is properly localized and that it is released in a controlled way [11]. In recent decades, several polymeric drug-loaded nanosystems have been developed for intratumoral drug delivery, including hydrogels [12], microparticles [13], nanoparticles [14], and nanofibers [15].

Magnetic nanoparticles (MNPs), a major class of nanoscale materials, have been investigated for decades in drug delivery systems because of their high magnetic responsiveness, biodegradability, biocompatibility, high delivery efficiency, and potential targeting function [16]. Under the control of external magnetic field, MNPs can direct therapeutic agents specifically to a diseased site, therefore reducing the dosage of the medication to minimize adverse drug effects [17]. However, the alteration of their surface in biological media and their behavior in vivo are two major limitations of MNPs drug delivery systems due to inevitable uptake by the reticuloendothelial system (RES) [18]. 

In recent years, many surface coating agents have been used to address the previous issue. Polyacrylic acid (PAA), a hydrophilic polymer that has functional groups capable both of bonding metal oxide nanoparticles to a pharmaceutical compound and of stably dispersing the metal oxide nanoparticles in water with an appropriate pH, was introduced in the surface engineering of nanoparticles [19]. Shuji et al. [20] have synthesized an efficient composite that contains a titanium dioxide core bonding to a pharmaceutical compound through PAA. Furthermore, our group has previously functionalized MNPs with PAA and investigated the adsorption capacity to amino acid [21]. Nevertheless, an MNPs drug loading system coated by PAA has never been reported. 

Herein, we aim to develop a MNPs drug delivery system with magnetic Fe_3_O_4_ cores and a shell of biocompatible PAA used to functionalize the MNPs. The physicochemical properties of the BLM-loaded MNPs (BLM-MNPs) were characterized in terms of morphology, size distribution, and drug loading content. Finally, in vitro release profiles of BLM from BLM-MNPs were examined in neutral environment. The results indicate that MNPs functionalized by PAA have high drug loading capacity and favorable release property for BLM.

## 2. Materials and Methods

### 2.1. Materials

FeCl_3_·6H_2_O, CH_3_COONa, ethylene glycol (EG), ETH, dimethylformamide (DMF), polyacrylic acid (MW: 3000), ethanol, isopropanol, 1-ethyl-3-(3-diethyl-aminopropyl) carbodiimide (EDAC), N-hydroxysuccinic acid, DMSO, PBS, and bleomycin A5 hydrochloride (BLM) were purchased from Harbin Bolai pharmaceutical company (Harbin PRC). Water used in the experiments was purified with the Millipore system. All chemicals are of reagent grade and used without further purification.

### 2.2. Preparation of Magnetic Fe_3_O_4_ Nanoparticles

FeCl_3_·6H_2_O (1 g) was dissolved in EG (20 mL) to form a clear solution, followed by the addition of NaAc (3 g) and ETH (10 mL). The mixture was stirred vigorously under ultrasonic vibration for 30 min and then sealed in a teflonlined stainless-steel autoclave. The autoclave was heated to and maintained at 200°C for 8 h and then cooled to room temperature. The black product was washed several times with water and pure alcohol. The magnetic and nonmagnetic particles were separated by using a magnet. Distilled water was added to prepare the sol of magnetic Fe_3_O_4_ nanoparticles with a solid content of 20%. 

### 2.3. Preparation of Magnetic Fe_3_O_4_ Nanoparticles Coated with Polyacrylic Acid

An aliquot of 0.75 mL of the magnetic Fe_3_O_4_ nanoparticle sol thus obtained was dispersed in 20 mL of the dimethylformamide (DMF), and 10 mL of DMF containing 0.3 g of polyacrylic acid dissolved therein was added and mixed by stirring. The solution was transferred into a teflonlined stainless-steel autoclave, and the reaction was performed at 150°C for 5 h. After the completion of the reaction, the autoclave was cooled until the temperature of the autoclave was 50°C or lower. The solution was removed, and then 60 mL of isopropanol was added. The resultant mixture was centrifuged at 4000 rpm for 20 minutes after letting it stand for 1 hour. The sediment was collected and washed with 70% ethanol, and then distilled water was added to prepare the polyacrylic acid-coated magnetic Fe_3_O_4_ nanoparticle dispersion liquid. 

### 2.4. Bleomycin Loading and Release Studies

Water was added to the magnetic Fe_3_O_4_ nanoparticles coated with polyacrylic acid to adjust the concentration of ferroferric oxide at 5% (w/v). 250 *μ*L of 800 mM 1-ethyl-3-(3-diethylaminopropyl) carbodiimide and 500 *μ*L of 100 mM N-hydroxysuccinic acid were added to 10 mL of the above adjusted solution, and a reaction was conducted with stirring at room temperature for 2 h. Then 0.8 mL of a solution of bleomycin hydrochloride dissolved in DMSO at 10 mg/mL was added, and a reaction was conducted with stirring at 4°C for 30 minutes.

**Figure 1:**
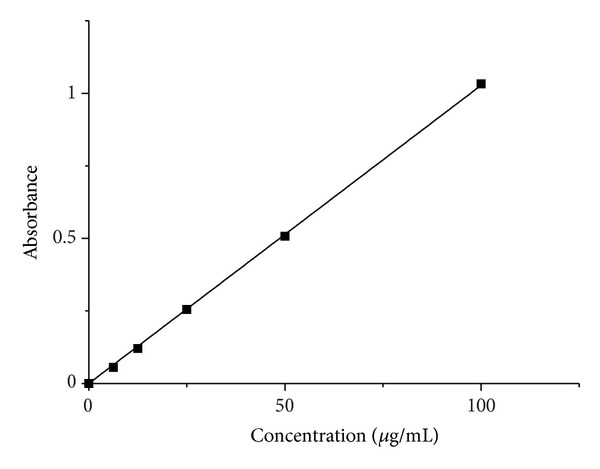
Bleomycin standard curve obtained using a UV spectrometer.

To determine the bleomycin loading and release rates, Bleomycin A5 hydrochloride (BLM) was dissolved in normal saline for use of evaluating the drug release properties of the Fe_3_O_4_ nanoparticles. 1.0 mg of bleomycin powder was dissolved in normal saline and then diluted to obtain the concentrations of 2, 4, 8, and 16 ug/mL. By using an ultraviolet (UV) spectrophotometer (Hitachi U4100 spectrophotometer), the absorbencies of the above concentrations were obtained at 291 nm with saline as the blank. These were used to establish a standard curve as shown in Figure 1. The tests were carried out at 37 ± 1°C and pH 7.4 ± 0.2.

 The amount of nonentrapped bleomycin in aqueous phase was determined using a UV spectrophotometer. The amount of bleomycin entrapped within the nanoparticles was calculated by the difference between the total amount of bleomycin used to prepare the nanoparticles and the amount of bleomycin present in the aqueous phase, using the following formula:

(1)  Loading  efficiency% =[(amount  of  loaded  drug  in  mg)(amount of the initially added drug in mg)]×100%.

The reaction products were then transferred to a dialysis tube and immersed in 10 mL of PBS buffer solution at 37°C with gentle shaking. At selected time intervals, buffer solution was taken for UV spectrometer and replaced with fresh buffer solution. The amount of bleomycin A5 released was determined by the UV spectrometer at 291 nm.

### 2.5. Characterization

Scanning electron microscopy (SEM) images were obtained by a scanning transmission electron microscope (S-520/INCA 300, Hitachi/Oxford, Japan) at an accelerating voltage of 20.00 kV. Infrared (IR) spectra were recorded with wavenumbers ranging from 4000 to 400 cm^−1^ by a Nicolet model 759 Fourier transform infrared (FT-IR) spectrometer using a KBr wafer. The magnetic properties (M-H curves) were measured at room temperature by the use of a Lake Shore 7404-Vibrating Sample Magnetometer (VSM). The optical absorbancies of the solutions of bleomycin mobilized and immobilized were measured using a Hitachi U4100 spectrophotometer.

### 2.6. Statistical Analysis

All data were analyzed with SPSS software (v 16.0; SPSS Inc, Chicago, IL). Results are presented as mean plus or minus standard deviation. The regression and correlation analysis was performed as deemed necessary.

## 3. Results and Discussion

### 3.1. SEM Image

**Figure 2:**
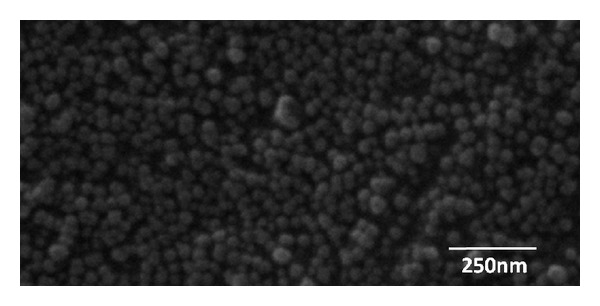
SEM image of naked magnetite nanoparticles.

Micrographs of Fe_3_O_4_ nanoparticles and PAA-coated Fe_3_O_4_ nanoparticles are shown in Figure 2. It can be observed that the magnetic Fe_3_O_4_ nanoparticles were well-shaped spheres with approximately 30 nm in diameter. Huang et al. [22] have found that the shape of the nanoparticles could affect their biodistribution, clearance, and biocompatibility in vivo. Nanoparticles which are more or less spherical show rapid clearance rates via urine and feces, thus reducing the risk of metal accumulation in human body. Moreover, spherical magnetite nanoparticles can offer a uniform surface area for coating and conjugation of targeting ligands or therapeutic agents. And the size of the nanoparticles can largely determine their half-life in the circulation. It has been reported that particles with a size range of 10–100 nm are considered to be optimum for the drug delivery [23] because they can easily escape the RES in the body with longer circulation times. Therefore, the nanoparticles we have synthesized are promising candidates for successful drug loading and delivery. 

### 3.2. Magnetic Properties

**Figure 3:**
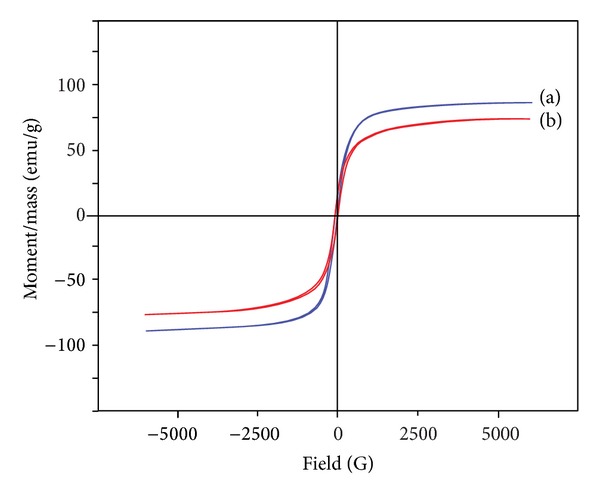
Magnetization curve of (a) naked Fe_3_O_4_ nanoparticles and (b) PAA-coated Fe_3_O_4_ nanoparticles.

Magnetization curves, as shown in Figure 3, were measured in powder samples of Fe_3_O_4_ nanoparticles at room temperature as applied magnetic field (Oe) changed from −5000 Oe to 5000 Oe. Both of the Fe_3_O_4_ nanoparticles and PAA-coated Fe_3_O_4_ nanoparticles exhibit negligible coercivity and remanence when the applied magnetic field was removed. The superparamagnetic behavior of the samples can make the particles easily disperse in the solution with inappreciable magnetic interactions between one another and avoid magnetic clustering. The saturation magnetizations obtained for Fe_3_O_4_ nanoparticles and PAA-coated Fe_3_O_4_ nanoparticles were 87 emu/g and 75 emu/g, respectively. The decrease of the saturation magnetization is due to the relatively lower density of magnetic components in the nanocomposites as polymer coated on the Fe_3_O_4_ nanoparticles. The synthetic strategy developed in this study offers a significant advantage in the saturation magnetization compared with other previous reports [21, 24], which is extremely important for the practical applications in biological and biomedical fields. 

### 3.3. Chemical Structure

**Figure 4:**
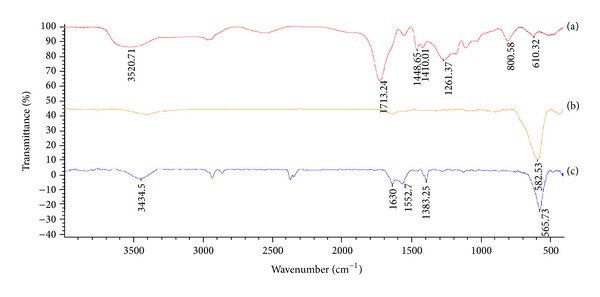
FT-IR spectra of (a) PAA, (b) naked Fe_3_O_4_ nanoparticles, and (c) PAA-coated Fe_3_O_4_ nanoparticles.

For the FTIR spectra (Figure 4) of the PAA, there is no stretching vibration of C=C group, indicating no residual monomer in PAA. As for the FTIR spectra of naked Fe_3_O_4_ nanoparticles and PAA-coated Fe_3_O_4_ nanoparticles, the peaks at 592 and 565 cm^−1^ are related to the Fe–O group and confirm the existence of Fe_3_O_4_. The peak at 1715 cm^−1^ for PAA and PAA-coated Fe_3_O_4_ nanoparticles was related to the C=O group. The IR spectrum of PAA-coated nanoparticles shows characteristic absorption bands at 1552 cm^−1^ and 1383 cm^−1^ which are assigned to C–O stretching and C=O stretching vibration, respectively. 

The hydrophilic polymers can be used to coat nanoparticles to extend their circulation time by reducing protein adsorption through the hydrophilicity or steric properties, which are thought to minimize uptake by RES cells [24]. PAA as one class of hydrophilic polymers can be bonded to the surface of the Fe_3_O_4_ nanoparticles by the reaction between the carboxyl group of the hydrophilic polymer and the hydroxyl group generated on the surface of the Fe_3_O_4_ nanoparticles by hydration of ferric oxide with water in the reaction system. Fe_3_O_4_ nanoparticles and the hydrophilic polymer are dispersed in dimethylformamide, and a hydrothermal reaction is performed at high temperature for several hours to bond them through an ester bond. The presence of infrared absorption around 1700 cm^−1^ which is the absorption band of the ester bond [20] manifests that they combined firmly after reaction. As the results have indicated, parts of carboxyl groups of PAA bond to the surface of the Fe_3_O_4_ nanoparticles while the others are exposed outside the shell. The latter can release the hydrogen ions under the condition of alkalescence, both stabilizing the particles dispersion in water and providing functional sites for the drug loading. 

### 3.4. In Vitro Loading and Release of BLM on the Fe_3_O_4_ Nanoparticles

Drug loading efficiency was 52.44 ± 0.11%, which was comparable with that of other drug-loaded nano-systems reported recently [25].

**Figure 5:**
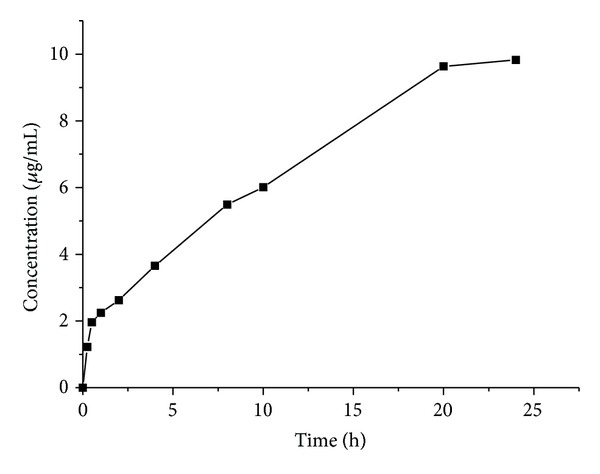
Drug release profile of BLM-MNPs measured at pH 7.5 in PBS buffer.

Figure 5 shows the release profile of BLM from BLM-loaded Fe_3_O_4_ nanoparticles in pH 7.5 over a time period of 24 h. Upon dialysis, approximately 98% of the drug was released within 24 h. The Fe_3_O_4_ nanoparticles immobilized with bleomycin have sustained drug release behavior. A small burst release of bleomycin occurred in the first 1 h, then following a rapid release about 22.4% of the total amount of drug. The following release of the drug was slower and approximately quantitative, while the release behavior slowed down after 20 h. The interesting release behaviors of BLM from BLM-MNPs could probably make them candidate for an effective functional material for magnet-controlled simultaneous cancer therapy in vivo, which is promising in biomedical field.

## 4. Conclusion

In summary, the superparamagnetic magnetite nanoparticles were synthesized by a simple solvothermal method. The saturation magnetization of the MNPs is as high as 87 emu/g, and the obtained Fe_3_O_4_ nanoparticles could be easily functionalized with carboxyl groups. The BLM loading and release behaviors of the BLM-MNPs were investigated. It was found that the loading efficiency of bleomycin to nanoparticles can reach 50% or higher. The drug release rates are also suitable for the drug delivery application, and most of the drug molecules incorporated could be immobilized to solution in 24 h. Hence, the PAA-coated Fe_3_O_4_ nanoparticles are very promising for the application in targeted delivery.

## References

[1] Moeller A, Ask K, Warburton D, Gauldie J, Kolb M (2008). The bleomycin animal model: a useful tool to investigate treatment options for idiopathic pulmonary fibrosis?. *International Journal of Biochemistry & Cell Biology*.

[2] Jin C, Jin Y, Wennerberg J, Rosenquist B, Mertens F (2008). Increased sensitivity to bleomycin in upper aerodigestive tract mucosa of head and neck squamous cell carcinoma patients. *Mutation Research*.

[3] Luo Q, Zhao F (2011). How to use bleomycin A5 for infantile maxillofacial haemangiomas: clinical evaluation of 82 consecutive cases. *Journal of Cranio-Maxillofacial Surgery*.

[4] Gobbi PG, Federico M (2012). What has happened to VBM (vinblastine, bleomycin, and methotrexate) chemotherapy for early-stage Hodgkin lymphoma?. *Critical Reviews in Oncology/Hematology*.

[5] Kurokawa S, Suda M, Okuda T (2010). Effect of inhaled KP-496, a novel dual antagonist of the cysteinyl leukotriene and thromboxane A2 receptors, on a bleomycin-induced pulmonary fibrosis model in mice. *Pulmonary Pharmacology & Therapeutics*.

[6] Azambuja E, Fleck JF, Batista RG, Menna Barreto SS (2005). Bleomycin lung toxicity: who are the patients with increased risk?. *Pulmonary Pharmacology & Therapeutics*.

[7] McGrath SE, Webb A, Walker-Bone K (2013). Bleomycin-induced Raynaud’s phenomenon after single-dose exposure: risk factors and treatment with intravenous iloprost infusion. *Journal of Clinical Oncology*.

[8] Adriane K, Huang J, Ding G, Chen J, Liu Y (2006). Self assembled magnetic PVP/PVA hydrogel microspheres; magnetic drug targeting of VX2 auricular tumours using pingyangmycin. *Journal of Drug Targeting*.

[9] Kavaz D, Odabaš S, Güven E, Demirbilek M, Denkbaš EB (2010). Bleomycin loaded magnetic chitosan nanoparticles as multifunctional nanocarriers. *Journal of Bioactive and Compatible Polymers*.

[10] Han B, Wang H-T, Liu H-Y, Hong H, Lv W, Shang Z-H (2010). Preparation of pingyangmycin PLGA microspheres and related *in vitro*/*in vivo* studies. *International Journal of Pharmaceutics*.

[11] Arruebo M, Fernández-Pacheco R, Ibarra MR, Santamaría J (2007). Magnetic nanoparticles for drug delivery. *Nano Today*.

[12] Díaz-Rodríguez P, Landin M (2012). Smart design of intratumoral thermosensitive *β*-lapachone hydrogels by Artificial Neural Networks. *International Journal of Pharmaceutics*.

[13] Chakravarthi SS, De S, Miller DW, Robinson DH (2010). Comparison of anti-tumor efficacy of paclitaxel delivered in nano- and microparticles. *International Journal of Pharmaceutics*.

[14] Nagahara LA, Ferrari M, Grodzinski P (2009). Nanofunctional materials in cancer research: challenges, novel methods, and emerging applications. *MRS Bulletin*.

[15] Wang H, Wei J, Yang C (2012). The inhibition of tumor growth and metastasis by self-assembled nanofibers of taxol. *Bioma Terials*.

[16] Hao R, Xing R, Xu Z, Hou Y, Goo S, Sun S (2010). Synthesis, functionalization, and biomedical applications of multifunctional magnetic nanoparticles. *Advanced Materials*.

[17] Sui Y, Cui Y, Nie Y, Xia G-M, Sun G-X, Han J-T (2012). Surface modification of magnetite nanoparticles using gluconic acid and their application in immobilized lipase. *Colloids and Surfaces B: Biointerfaces*.

[18] Jain TK, Foy SP, Erokwu B, Dimitrijevic S, Flask CA, Labhasetwar V (2009). Magnetic resonance imaging of multifunctional pluronic stabilized iron-oxide nanoparticles in tumor-bearing mice. *Biomaterials*.

[19] Ge J, Hu Y, Yin Y (2007). Highly tunable superparamagnetic colloidal photonic crystals. *Angewandte Chemie*.

[20] Shuji S, Koki K, Yumi O, Toshiaki B, Yoshinobu K Therapeutic method of administering pharmaceutical titanium dioxide composite and light irradiation. http://www.faqs.org/patents/app/20110014245#b#ixzz2RzoIfzXF.

[21] Xu Y, Zhuang L, Lin H, Shen H, Li JW (2013). Preparation and characterization of polyacrylic acid coated magnetite nanoparticles functionalized with amino acids. *Thin Solid Films*.

[22] Huang X, Li L, Liu T (2011). The shape effect of mesoporous silica nanoparticles on biodistribution, clearance, and biocompatibility *in vivo*. *ACS Nano*.

[23] Gupta AK, Wells S (2004). Surface-modified superparamagnetic nanoparticles for drug delivery: preparation, characterization, and cytotoxicity studies. *IEEE Transactions on Nanobioscience*.

[24] Milton Harris J, Chess RB (2003). Effect of pegylation on pharmaceuticals. *Nature Reviews Drug Discovery*.

[25] Lv Y, Ding G, Zhai J, Guo Y, Nie G, Xu L (2013). A superparamagnetic Fe_3_O_4_-loaded polymeric nanocarrier for targeted delivery of evodiamine with enhanced antitumor efficacy. *Colloids and Surfaces B: Biointerfaces*.

